# Case Report: Unclassified Renal Cell Carcinoma With Medullary Phenotype and *SMARCB1/INI1* Deficiency, Broadening the Spectrum of Medullary Carcinoma

**DOI:** 10.3389/fmed.2022.835599

**Published:** 2022-02-07

**Authors:** Marina Valeri, Miriam Cieri, Grazia Maria Elefante, Camilla De Carlo, Noemi Rudini, Giovanni Lughezzani, Nicolò Maria Buffi, Luigi Maria Terracciano, Piergiuseppe Colombo

**Affiliations:** ^1^Department of Biomedical Sciences, Humanitas University, Pieve Emanuele, Italy; ^2^Department of Pathology, Istituto di Ricovero e Cura a Carattere Scientifico (IRCCS) Humanitas Clinical and Research Hospital, Rozzano, Italy; ^3^Department of Urology, Istituto di Ricovero e Cura a Carattere Scientifico (IRCCS) Humanitas Clinical and Research Hospital, Rozzano, Italy

**Keywords:** renal medullary carcinoma, renal cell carcinoma unclassified with medullary phenotype, *SMARCB1/INI1*, sickle cell trait, kidney, case report

## Abstract

Renal medullary carcinoma (RMC) is a rare entity with poor prognosis bearing inactivating genomic alterations in *SMARCB1/INI1* resulting in the loss of expression of INI1 and occurring in young patients with sickle cell trait or sickle cell disease. Recently, rare examples with histological characteristics of RMC have been described in older patients without hemoglobinopathies and provisionally termed “Renal cell carcinoma unclassified with medullary phenotype” (RCCU-MP). Fluorescence *in situ* Hybridization (FISH) can detect alterations in *SMARCB1/INI1* consisting mostly in inactivating translocation of one allele and deletion of the second. To date, only seven further cases of RCCU-MP have been described in the literature. Here we report the second Italian case of RCCU-MP, a 62-year-old man presenting with persistent dull back pain and incidentally discovering a 13 cm mass in the right kidney. The nomenclature of this entity is still debated and might be updated as a variant of medullary carcinoma in the upcoming WHO classification. In the meantime, we encourage awareness of these extraordinarily rare neoplasms with poor outcomes.

## Introduction

Renal medullary carcinoma (RMC) is an aggressive neoplasm accounting for <0.5% of renal cell carcinomas (RCC) (1, 2). This rare entity occurs mainly in the third decade of life and almost all (> 95%) patients have sickle cell trait, sickle cell disease, or associated hemoglobinopathies (2). RMC is characterized by inactivating genomic alterations in *SMARCB1/INI1*, a tumor suppressor gene (3), resulting in the loss of immunohistochemical expression of INI1 in all cases (4–6). Recently, exceedingly rare tumors sharing morpho-phenotypic features with RMC, but occurring in older patients without hemoglobinopathy have been reported in a few case reports and our recent small case series (7–9) and provisionally termed “RCC unclassified with medullary phenotype” (RCCU-MP) (7, 10). Alterations in *SMARCB1/INI1* can be detected by Fluorescence *in situ* Hybridization (FISH). Almost 85% of patients with RMC show a genetic loss, most commonly due to inactivating translocation of one *SMARCB1* allele and deletion of the second allele. Less frequently, deletion of both *SMARCB1* alleles, deletion of one *SMARCB1* allele and inDel of the second *SMARCB1* allele, or deletion of one *SMARCB1* allele and truncating non-sense mutation of the second *SMARCB1* allele might occur (11). Here we report the second Italian case of RCCU-MP, a fascinating entity whose definition might be further clarified by the new upcoming WHO classification.

## Case Description

**Figure 1:**
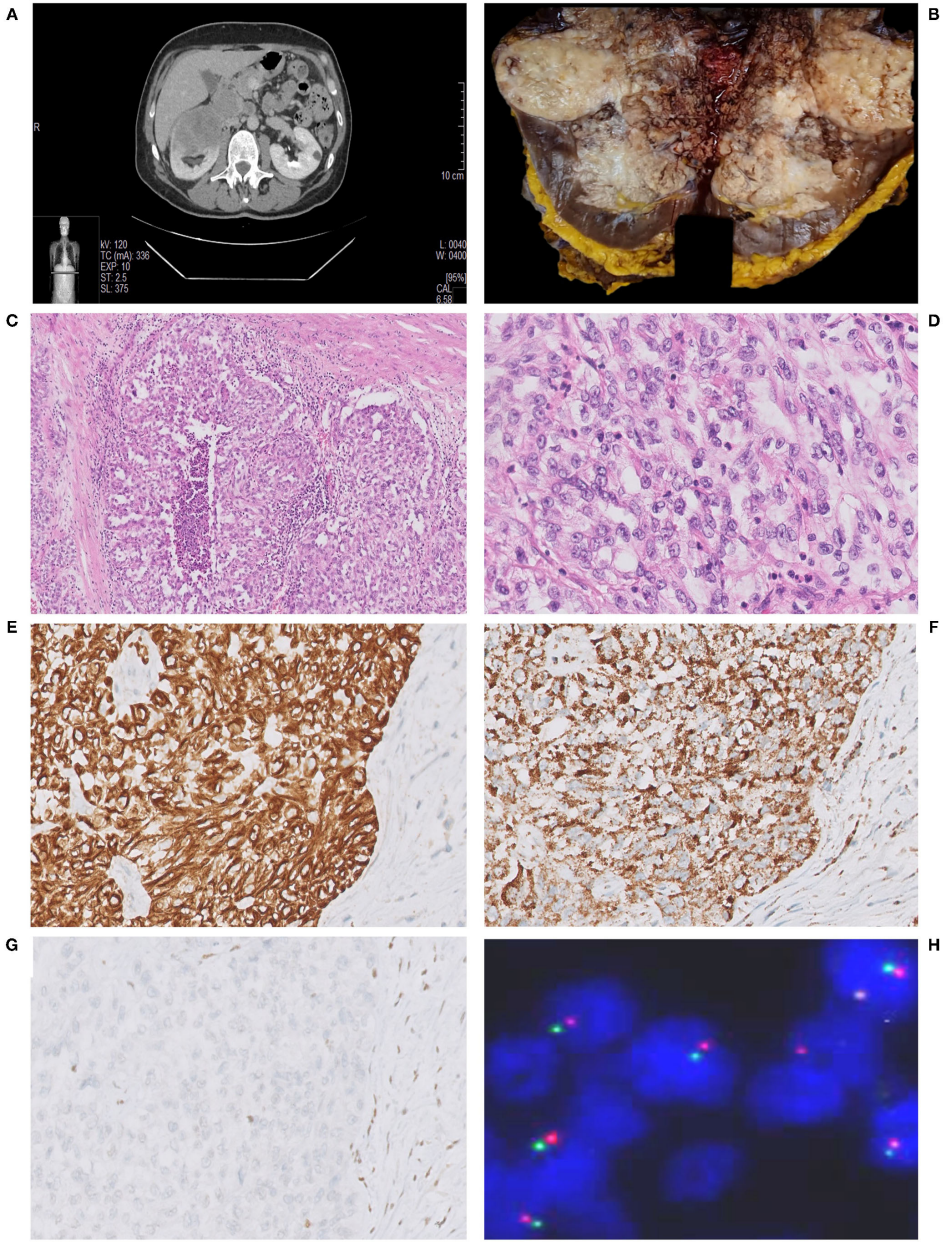
Abdominal CT scan showing a bulky renal mass in the upper pole of the right kidney **(A)**. Grossly, renal parenchyma was partially replaced by a whitish, solid, and necrotic mass **(B)**. Histological view showing a solid proliferation of eosinophilic, highly pleomorphic, epithelioid cells, with nested growth pattern and focal pseudo-glandular differentiation, associated with desmoplastic stromal response. Multiple foci of necrosis were present (30% of tumor). Notably, many cells had a high nuclear-cytoplasmic ratio with vesicular nuclei and prominent nucleoli **(C,D)**. Immunohistochemically, the tumor was CK7+ in almost all cells **(E)** and fumarate hydratase (FH) expression was retained **(F)**; neoplastic cells showed the characteristic loss of INI1 **(G)**. Fluorescence *in situ* hybridization (FISH) with SPEC *SMARCB1/22q12* Dual Color Probe detected, in a representative tumoral area, a loss of one *SMARCB1* allele (single green and orange signal per cell) in almost half of the cells **(H)**.

A 62-year-old Italian man presented with persistent dull back pain. The patient was on anticoagulant therapy for recent pulmonary thromboembolism, had no previous history of malignancy or surgery, and presented no comorbidities. Ultrasound abdominal examination detected a renal mass, confirmed by a CT scan that documented a 13 × 10.8 × 5 cm solid tumor in the upper pole of the right kidney (Figure 1A), renal vein, and inferior vena cava thrombosis.

Right radical nephrectomy with cavotomy, thrombectomy, and regional lymphadenectomy was performed.

At gross examination, the tumor presented as a whitish, extensively necrotic mass of 13.1 cm in greatest dimension, infiltrating into the perinephric and renal sinus adipose tissue; neoplastic thrombosis was confirmed (Figure 1B). Incidentally, a metastatic nodule was documented in the ipsilateral adrenal gland.

Histologically, the tumor consisted of a proliferation of epithelioid cells, with enlarged and pleomorphic nuclei and eosinophilic cytoplasm, showing predominant solid and nested growth pattern, focally glandular, infiltrative borders, and extensive replacement of renal medulla (Figures 1C,D). The neoplasm was associated with desmoplastic stromal response, inflammatory lymphocytic infiltrate, and multiple foci of necrosis (~30% of the tumor). One metastatic hilar lymph node was observed. Non-neoplastic kidney showed chronic interstitial nephritis and mild glomerulosclerosis. Histological features, together with medullary involvement, prompted us to the hypothesis of medullary carcinoma.

The tumor cells showed an immunophenotype specific of the proximal renal tubule, with immunopositivity for cytokeratin 7, PAX8, and FH (retained), and absence of GATA3 and OCT3/4. Focal immunoreactivity for CA IX and Racemase was found. Expression of INI1 was lost, as expected in the suspicion of RMC (Figures 1E–G). Blood tests did not show evidence of sickle cell trait, sickle cell disease, or any other hemoglobinopathy, therefore the diagnosis of RCCU-MP was made. To detect *SMARCB1/INI1* alterations, FISH was performed on paraffin sections of both tumoral areas and adjacent normal tissue using a commercial SPEC *SMARCB1/22q12* Dual Color Probe (Zyto*Light*®, according to the manufacturer's protocol). Loss of one *SMARCB1* allele was found in 42% of cells in the tumoral area (hemizygous deletion) (Figure 1H). Six months later, the patient experienced mediastinal nodal, hepatic, and bone metastases. Therefore, systemic therapy (Pembrolizumab + Axitinib and Radiotherapy on bone lesions) was administered. After 8 months of follow-up, the patient was alive with the disease.

## Discussion

In this study, we described the eighth case reported so far of RCCU-MP (7–9). The provisional diagnostic terminology of “RCCU-MP” has been recently proposed by international genitourinary pathologists for extraordinarily rare tumors with morphological and phenotypical characteristics of RMC but without sickle cell trait nor sickle cell disease (10, 12). However, the definite designation of these neoplasms is still debated (2).

In the past, this entity has been mislabeled as other RCC subtypes, such as unclassified RCC or Collecting duct carcinoma (CDC). Therefore, it has been possibly under-reported in the literature. Indeed, RCCU-MP is often a challenging diagnosis. In the absence of hemoglobinopathies, the differential diagnosis of RCCU-MP comprises CDC (HMWK+, OCT3/4-, INI1+), upper tract urothelial carcinoma (UTUC) (GATA3+, p63+, OCT3/4-, INI1+), Fumarate-Hydratase deficient RCC (FH-d RCC) (FH-, OCT3/4-, INI1+), ALK-rearranged RCC (ALK+, INI1+), and metastatic carcinoma (2). Rarely, some rhabdoid features might raise the differential diagnosis with rhabdoid tumors of the kidney (9).

Genes related to hypoxia have been advocated in the pathogenesis of *SMARCB1* abnormalities in RMC, triggered by red blood cell sickling and subsequent ischemia. *SMARCB1* has been suggested to be located in a hotspot region for *de novo* mutations susceptible to hypoxic stress mediated by peculiar medullary microvascular physiology (13). Given the overlapping features between RMC and RCCU-MP, Sirohi et al. recently suggested that a genetic predisposition unrelated to hemoglobinopathies might lead to *SMARCB1* abnormalities mediated by the same vascular mechanisms (14). Although interesting, this hypothesis needs further validation.

To the best of our knowledge, only seven further cases of RCCU-MP have been systematically collected and reported in the literature (7–9), whose clinical features are summarized in Table 1. The patients were mostly Caucasian and men, with a mean age of 52 years, and almost all cases were locally advanced (pT3) with distant metastases. Interestingly, in only one case genetic alterations were investigated, with a negative result (9). All cases were centered in the renal medulla; the main morphological pattern was solid, either nested or cord-like, often with necrosis and diffuse polymorphism. Four cases showed rhabdoid features while desmoplasia and inflammation were documented in almost all patients. Morphological features are summarized in Table 2. All cases were INI1-(lost), PAX8+ and FH+(retained), 4/5 cases were CK7+, and 5/7 cases were OCT3/4+.

**Table 1:** Clinical and pathologic data for RCCU-MP cases.

	**References**	**Age (y)**	**Sex**	**Race** **/Country**	**Size (cm)**	**Side**	**pTNM**	**Sickle trait**	**Nodal status**	**Metastasis**	**Progression**	**Status**	**FU (mo)**	**Therapy**	***SMARCB1/ INI1***
Case 1	Sirohi et al. (7)	39	M	C/US	19	R	pT3cN1M1	HBE (-), HbS screen (-)	Aortocaval, supraclavicular	Adrenal, lung, bone, liver, mediast.	*Presentation*, 5*mo*	DOD	27	RN, CT, RT	IHC (lost)
Case 2	Sirohi et al. (7)	71	M	C/Italy	6.5	L	pT3aN1M1	HBE (-)	Hilar	Pelvic bone (5 cm)	*Presentation*	DOD	3	RN, CT	IHC (lost)
Case 3	Sirohi et al. (7)	58	M	C/US	3.4	L	pT3aN1Mx	No history, no anemia >9 y	Periaortic, paracaval	Peritoneum and bone	2*mo*	DOD	3	RN	IHC (lost)
Case 4	Sirohi et al. (7)	24	F	C/US	5.5	L	pT3aN0Mx	HBE (-)	No	None	*None*	NED	12	RN	IHC (lost)
Case 5	Sirohi et al. (7)	30	M	A/US	4.5	R	Biopsy only	HBE (-)	Retroperitoneum	Lung, bone, and liver	3*mo*	DOD	9	CT	IHC (lost)
Case 6	Lai et al. (8)	76	M	C/US	6.3	R	pT3aNxM1	No history	No	Lung	3*mo*	AWD	3	RN, CT	IHC (lost)
Case 7	Tsuzuki et al. (9)	63	M	A/Japan	4.3	L	pT3aN0	HbS solubility testing (-)	No	None	7*mo*	NED	4	RN, CT	IHC (lost); FISH (retained); seq. (wild-type)
Case 8	Current	62	M	C/Italy	13.1	R	pT3bN1M1	Hbs screen (-)	Hilar, subcarenal	Adrenal, liver, bone	6*mo*	AWD	8	RN, CT, RT	IHC (lost); FISH (lost)

*M, male; F, female; R, right; L, left; C, Caucasian; A, asian; LN, lymph node; HBE, hemoglobin electrophoresis; HbS, hemoglobin sickle; FU, follow-up; DOD, dead of disease; AWD, alive with disease; NED, no evidence of disease; RN, radical nephrectomy; CT, chemotherapy; RT, radiotherapy; IHC, immunohistochemistry; FISH, fluorescence in situ hybridization*.

**Table 2:** Morphologic features.

	**References**	**Capsule**	**Margins**	**Location**	**Necrosis**	**Renal Vein Invasion**	**ISUP Grade**	**Main pattern**	**Other Patterns**	**Specific morphology**	**Dysplasia/ Ca *in situ***	**Desmoplasia**	**Inflammation**	**HLRCC-like Nuclei**
Case 1	Sirohi et al. (7)	No	Infiltrative	Cortex medulla	Yes	Yes	4	Solid, nested, cord-like	Tubular/ tubulopapillary	Rhabdoid	*Yes*	Fibroblastic	Acute	Focal
Case 2	Sirohi et al. (7)	No	Infiltrative	Cortex medulla	Yes	No	4	Solid	Infiltrative glandular pattern, focal cribriform	Sarcomatoid, focal giant cell	*No*	Fibroblastic	Acute and lymphoplasmacytic	No
Case 3	Sirohi et al. (7)	Focally	Polycyclic	Medulla	Yes	No	4	Solid, nested, cord-like	Tubular/ tubulopapillary	Rhabdoid	*No*	Fibroblastic	Lymphocytic	Focal
Case 4	Sirohi et al. (7)	No	Infiltrative	Medulla	No	No	3	Solid, nested, cord-like	Tubular/ tubulopapillary, reticular, cribriform	Rhabdoid	*No*	Sclerosis	Lymphocytic	No
Case 5	Sirohi et al. (7)	Biopsy	Biopsy	Biopsy	No	Biopsy	3	Infiltrative glandular	Reticular, non-glandular	NA	*Yes*	Fibromyxoid	Lymphocytic	No
Case 6	Lai et al. (8)	NA	Infiltrative	Cortex medulla	NA	No	3	Nested	Single glands and cribriform with cystic changes	NA	*NA*	Yes, nos	Acute	NA
Case 7	Tsuzuki et al. (9)	No	Infiltrative	Cortex medulla	Yes	No	3	Nested	Tubular, cord-like	Rhabdoid	*NA*	NA	Lymphocytic	NA
Case 8	Current	No	Infiltrative	Cortex medulla	Yes	Yes	4	Solid and nested	Glandular	No	*No*	Fibroblastic	Lymphocytic	No

*NA, not assessed; ISUP, international society of urological pathology; HLRCC, hereditary leiomyomatosis and renal cell cancer; nos, not otherwise specified*.

This is the eighth case of RCCU-MP reported so far. The current classification of this rare entity is still debated: RCCU-MP and RMC share both morphological and phenotypical features so they might be regarded as variants of the same disease in the next future.

Waiting for the new WHO classification, awareness of the diagnosis of these rare entities should be encouraged since they identify patients with poor prognoses and might reveal unacknowledged hemoglobinopathies.

## Data Availability Statement

The original contributions presented in the study are included in the article, further inquiries can be directed to the corresponding author/s.

## Ethics Statement

Written consent was acquired from the patient.

## Author Contributions

PC and MV conceived, designed the study, wrote, and revised the final manuscript. LT, MC, GE, and CD reviewed the histological slides and revised the final manuscript. NR performed the FISH analysis. GL and NB performed surgery and provided clinical data. All authors contributed to the article and approved the submitted version.

## Conflict of Interest

The authors declare that the research was conducted in the absence of any commercial or financial relationships that could be construed as a potential conflict of interest.

## Publisher's Note

All claims expressed in this article are solely those of the authors and do not necessarily represent those of their affiliated organizations, or those of the publisher, the editors and the reviewers. Any product that may be evaluated in this article, or claim that may be made by its manufacturer, is not guaranteed or endorsed by the publisher.
